# Safety Evaluation for Fabricated Small Box Girder Bridges Based on Fuzzy Analytic Hierarchy Process and Monitoring Data

**DOI:** 10.3390/s24144592

**Published:** 2024-07-15

**Authors:** Hongyin Yang, Liangwei Jiang, Feng Xu, Jianfeng Gu, Zhongtao Ye, Ya Peng, Zhangjun Liu, Renhui Cheng

**Affiliations:** 1 School of Civil Engineering and Architecture, Wuhan Institute of Technology, Wuhan 430073, China; 2 Hubei Provincial Engineering Research Center for Green Civil Engineering Materials and Structures, Wuhan 430073, China; 3 State Key Laboratory of Bridge Intelligent and Green Construction, Wuhan 430034, China

**Keywords:** safety evaluation, structural health monitoring, fabricated small box girder bridge, temperature field, fuzzy analytic hierarchy process

## Abstract

During the operation of fabricated small box girder bridges, which face safety issues such as structural degradation and failure, there is an urgent need to propose a safety evaluation method to cope with the possible risks. This article quantitatively evaluates the safety state of a fabricated small box girder bridge in Wuhan City based on Fuzzy Analytic Hierarchy Process (FAHP) and structural health monitoring (SHM) data. Firstly, the FAHP model is established, and stress, deformation, and temperature are selected as evaluation factors. The safety thresholds of stress and deformation are determined by combining the industry specifications and the historical statistical patterns of the massive SHM data. The temperature field of the bridge is simulated and analyzed by combining ANSYS, HYPERMESH, and TAITHREM, and the most unfavorable temperature gradient is determined as a threshold for the safety evaluation. Finally, the scores of indexes of the bridge are determined based on the measured SHM data, which in turn provides a quantitative description of the safety state. The results show that the thresholds determined by the joint industry specifications and the massive SHM data are reasonable; the temperature field simulation model established in this article is consistent with the measured results, and can accurately determine the temperature gradient of the bridge. The safety evaluation result from the FAHP model is the same as the field test results, which verifies the effectiveness and applicability of the proposed method to actual bridge projects.

## 1. Introduction

Fabricated small box girders have significant advantages such as being lightweight, having high torsional stiffness, uniform lateral distribution of live loads, and convenience for transportation and installation, which are widely used in small-span bridges [1,2]. However, during the operation of the bridges, due to external environmental factors such as wind [3], temperature [4,5], and earthquakes [6]; overload operation; and structural performance degradation, the existing bridges inevitably suffer from various damages such as cracking and spalling of concrete in varying degrees, and corrosion of steel reinforcement [7]. Therefore, it is necessary to conduct regular bridge safety inspections and safety evaluations, which are conducive to the timely detection of potential safety hazards and the adoption of appropriate reinforcement measures, thereby ensuring the safe operation of bridges [8].

With the development of structural health monitoring (SHM) technology, more and more bridges are equipped with SHM systems during operation, which allows bridge owners and researchers to achieve remote real-time monitoring and safety evaluation through SHM data [9,10]. A decision-making basis for bridge safety evaluation can be provided by analyzing the change patterns of the massive SHM data and extracting the components reflecting the structural performance of bridges [11,12,13]. Therefore, in order to accurately evaluate the safety state of bridges, proposing reasonable and effective safety evaluation methods based on the actual state of bridges is necessary.

To this end, much effort has been devoted to studying safety evaluation methods of bridges. Melhem et al. [14] found a model suitable for bridge condition evaluation based on the priority feature vector. Sasmal et al. [15] proposed a fuzzy logic theory method for state evaluation for prestressed concrete bridges. Seo et al. [16] pointed out that bridge safety monitoring should focus on damage identification, residual bearing capacity evaluation, and structural residual life evaluation. Bayane et al. [17] proposed a method to evaluate the fatigue safety of concrete bridge decks using SHM data. Based on temperature and strain monitoring data of concrete bridges, Obrien et al. [18] proposed a method for bridge safety evaluation using temperature information and damage indicators. Zhou et al. [19], reviewed the application of SHM systems in bridge safety evaluation and pointed out that new methods are needed for the rational application of SHM data. Fu et al. [20] used hierarchical analysis (AHP) to quantify the safety evaluation of bridges during the construction period and noted that multi-source data can provide important data support for bridge safety evaluation. However, how to determine reasonable safety evaluation thresholds and select reliable evaluation methods for bridges are topics that need further research, at the same time, few previous studies have considered temperature gradient as an influencing factor for fabricated small box girder bridges.

One of the keys to bridge safety evaluation is the selection of the threshold of the evaluation index. The safety evaluation methods of bridge structures already have a specific theoretical basis and technical support. Most current safety evaluation methods for bridge structures are based solely on relevant industry specifications or calculated results of finite element models (FEMs), and thresholds are selected based on theoretical approaches [21,22,23]. Because of the complexity of the bridges and the impact of the external environment, evaluation results based solely on theoretical thresholds cannot thoroughly reflect the actual state of bridges [24,25]. Therefore, in order to achieve the effective application of bridge safety evaluation in engineering practice, it is necessary to consider various factors such as SHM data, industry specifications, and theories, and then select reasonable thresholds.

Another key to bridge safety evaluation is the identification of evaluation models. The Fuzzy Analytic Hierarchy Process (FAHP) is a method that extends the traditional AHP to address uncertainties and vagueness in decision-making. The FAHP allows decision-makers to incorporate qualitative and imprecise judgments by using linguistic variables rather than precise numerical values [26,27]. Determination of affiliation is one of the keys of the FAHP [28], and the affiliation is usually determined by expert scoring and membership functions. The FHAP model is often divided into the target layer, factor layer, and index layer. For bridge safety evaluation, the evaluation factors are often selected as stress, deformation, and environment.

The evaluation indexes corresponding to the environmental factor need to be focused on, and it is necessary to analyze the temperature gradients of fabricated concrete small box girder bridges [29,30,31]. However, there are discrepancies between the temperature gradients given in the industry specifications and the measured values, especially at the bottom of the concrete box girders. The accurate determination of the temperature gradient threshold is a challenge that must be addressed. Tao et al. [32] regarded the long-term temperature field of the bridge as a stochastic process and modeled the temperature field by simulating a non-Gaussian stochastic process, which achieved a long-term temperature field simulation based on limited measured data, but the method did not extend to a three-dimensional space. Therefore, it is necessary to model the temperature field of an entire bridge based on long-term SHM data.

In this article, a fabricated small box girder bridge in Wuhan City is taken as the engineering background, and its safety state is evaluated based on the FAHP and SHM data. The FAHP model is divided into the target layer, factor layer, and index layer. The stress, deflection, pier-girder relative displacement, and temperature gradient are selected as indexes, and the weights of each index are determined by expert scoring. The thresholds of stress, deflection, and pier-girder relative displacement are determined by combining relevant industry specifications and historical statistical patterns of the SHM data. The spatio-temporal FEM of the temperature field of the bridge is simulated jointly with ANSYS, HYPERMESH, and TAITHREM, the accuracy of the FEM is verified by the SHM measured data, and then the calculated most unfavorable temperature gradient of the bridge cross-section is used as a threshold for safety evaluation. The scores of indexes of the bridge are determined based on the measured SHM data, which in turn provides a quantitative description of the safety state.

## 2. Theory

### 2.1. Fuzzy Analytic Hierarchy Process

The FAHP decomposes the problem hierarchically, constructing a bottom-up ladder based on the nature and general objectives of the multi-objective evaluation problem. The specific steps are as follows:

#### 2.1.1. Building a Hierarchical Structure

The multi-objective decision-making problem is divided according to the hierarchical structure into target, factor, and index layers in turn.

#### 2.1.2. Establishment of Fuzzy Judgement Matrix

The fuzzy judgment matrix **P** can be obtained by the 9-scale method [33], which quantitatively describes the relative importance of the indexes by comparing them two by two and determines the affiliation between the indexes. **P** can be expressed as follows:

(1) $$P={\left(p_{ij}\right)}_{n\times n}$$

#### 2.1.3. Calculate the Weight Vector

(2) $$W_{i}=\frac{\sum_{j=1}^{n}p_{ij}+\frac{n}{2}-1}{n(n-1)},i=1,2,\ldots ,n$$

where $\sum_{j=1}^{n}p_{ij}$ is the sum of the elements of row *i*.

#### 2.1.4. Calculate the Identity Matrix

Suppose $W={\left(W_{1},W_{2},\ldots ,W_{n}\right)}^{T}$ is the weight vector for the different indexes, where $\sum_{i=1}^{n}W_{i}=1,W_{i}\geq 0(i=1,2,\ldots ,n)$, and suppose that

(3) $$W_{i,j}=\frac{W_{i}}{W_{i}+W_{j}}(\forall i,j=1,2,\ldots ,n)$$

then the identity matrix W^*^ can be expressed as follows:

(4) $$W^{*}={\left(W_{ij}\right)}_{n\times n}$$

#### 2.1.5. Consistency Test

The consistency test is designed to avoid mistakes in expert scoring. The calculation of the compatibility index *I* (**P**, W^*^) for the **P** and W^*^, and *I* can be expressed as follows:

(5) $$I(P,W^{*})=\frac{1}{n^{2}}\sum_{i=1}^{n}\sum_{j=1}^{n}\left|p_{ij}+W_{ji}-1\right|$$

If I≤α, the consistency test is considered to be passed; a smaller α indicates that the decision maker has higher requirements for the judgment matrix, and also indicates that the expert scoring is more reasonable. Generally, α is equal to 0.1.

#### 2.1.6. Comprehensive Evaluation

By calculating the comprehensive evaluation indicator *Q*, the impacts of all indexes are combined to obtain a quantitative evaluation. *Q* can be expressed as follows:

(6) $$Q=W_{T}\cdot R$$

where **W**_T_ is the total weight of different indexes, and **R** is the evaluation matrix, which is determined according to the measured signals of the bridge SHM system in this article.

### 2.2. Threshold Determination

In the process of safety evaluation for bridges, it is essential to set appropriate thresholds. Based on the selected bridge safety evaluation indexes, this article determines the safety evaluation thresholds from the following three aspects:

#### 2.2.1. Relevant Industry Specification Thresholds

Specification thresholds are set by relevant national or regional standards and protocols, and bridge indexes should not far exceed the industry specification thresholds or else there is a safety risk.

#### 2.2.2. Historical Data Statistics Threshold

More and more, bridges have been equipped with SHM systems. The signals collected by the SHM systems are statistically analyzed, thresholds are set with a 95% guarantee rate, and the results are incorporated into the safety evaluation system, which is regularly updated. Determining the thresholds based on the historical statistical values of the SHM data, the actual state of the bridge can be dynamically evaluated.

#### 2.2.3. Temperature Gradient Threshold

Bridges in the external environment for a long time exchange heat with the environment through heat conduction, heat convection, heat radiation, and other ways, but different heat exchange rates will lead to the formation of uneven temperature distribution and temperature gradient.

The simulation of the temperature field of the bridge is an important part of this article. TAITHERM 2020.2 is a professional thermal simulation and analysis tool, which was chosen to simulate the temperature field of the small box girder bridge in this article. However, it is only suitable for TAITHERM to construct simple standard models and system combinations, and it is difficult for TAITHERM to model complex geometric models; the APDL program of ANSYS can solve this problem perfectly. Since TAITHERM is not compatible with the file format of ANSYS, HYPERMESH was used to partition the mesh, and then TAITHREM was used to calculate and analyze the partitioned mesh. The specific steps for modeling the spatio-temporal FEM can be summarized as follows:

(1) Use ANSYS 2020 R1 to build the geometric model of the assembled small box girder according to the design drawings;
(2) Import the geometric model into HYPERMESH to divide the mesh;
(3) Introduce the meshed geometric model into TAITHERM, then set the thermal boundary conditions and import the meteorological data including temperature, humidity, wind speed, solar radiation, etc., of the bridge site to achieve the long-term thermodynamic analysis of the bridge.

Environmental change is an important factor affecting the safety state of bridges; the bridge temperature gradient was reasonably analyzed by the spatio-temporal FEM and the temperature gradient threshold was determined to provide reasonable data support for the subsequent safety evaluation.

**Figure 1:**
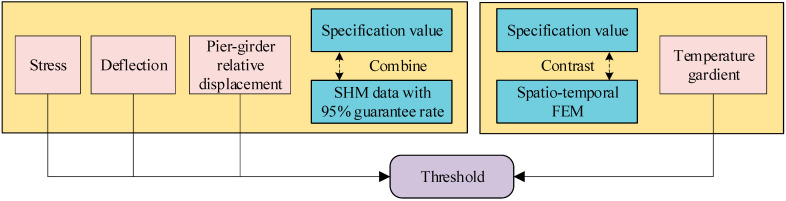
Determination method of the thresholds.

The threshold determination method in this article is shown in Figure 1.

### 2.3. Flowchart of the Proposed Safety Evaluation Method

In order to evaluate the safety state of a fabricated small box girder bridge, this article proposes a safety evaluation method based on the FAHP and SHM data, which can be divided into the following five steps:

1. Based on the bridge safety evaluation target, the FAHP is decomposed into the target layer, factor layer, and index layer. The judgment matrix is determined by the 9-scale method and consistency tests are performed.
2. The SHM data is preprocessed to remove high-frequency noise.
3. Stress, deformation, and environmental factors are selected as factor layers. Stress, deflection, pier-girder relative displacement, and temperature gradient are correspondingly selected as index layers. The SHM data of the fabricated small box girder bridge is processed to analyze the statistical patterns, and combined with relevant industry specifications, the thresholds for stress, as well as deformation, are determined.
4. The spatio-temporal FEM of the temperature field of the bridge is determined through ANSYS, HYPERMESH, and TAITHREM, the most unfavorable temperature gradient is verified, and the threshold of the temperature gradient is determined.
5. Based on the SHM data and relevant thresholds, the evaluation matrix of the bridge safety state is obtained, and the evaluation results are calculated with the FAHP model to quantitatively describe the bridge safety state.

**Figure 2:**
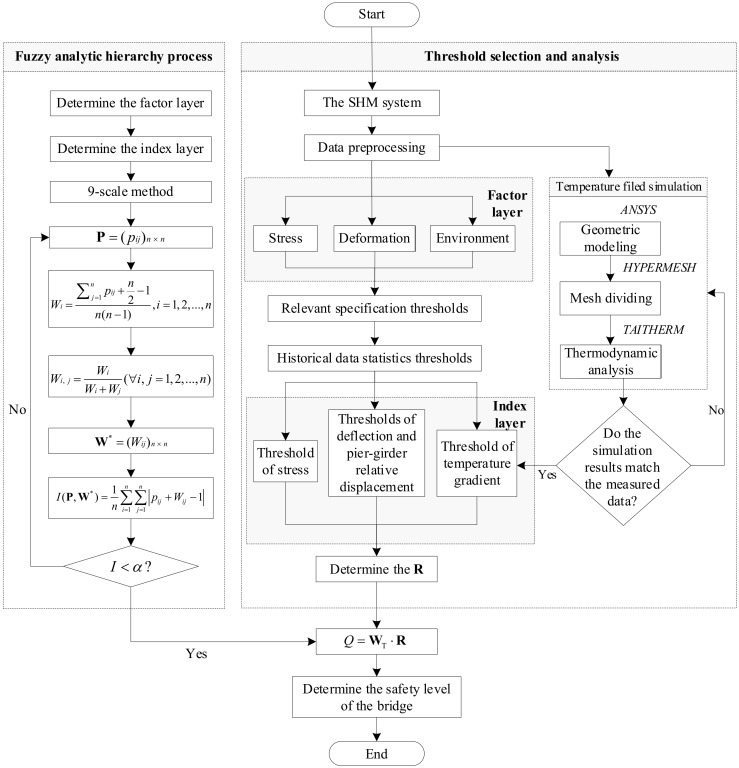
Flowchart of the bridge safety evaluation method.

The flowchart of the bridge safety evaluation method is shown in Figure 2.

## 3. Case Study

### 3.1. Engineering Background

In this article, a small fabricated concrete box girder bridge with an equal cross-section in Wuhan City was taken as the engineering background. The axis direction of the bridge is the east-west direction, and the span layout is 5 × 25 m; the width of the top plate of the small box girder is 13.25 m; the width of the left and right flanges is 0.6 m. A total of 10 cm of a C50 concrete leveling layer and 9 cm of an asphalt concrete paving layer are laid above the top plate; the thickness of the top, bottom, and web plates of a small box girder bridge is 10 cm, 16 cm, and 30 cm, respectively. 

**Figure 3:**
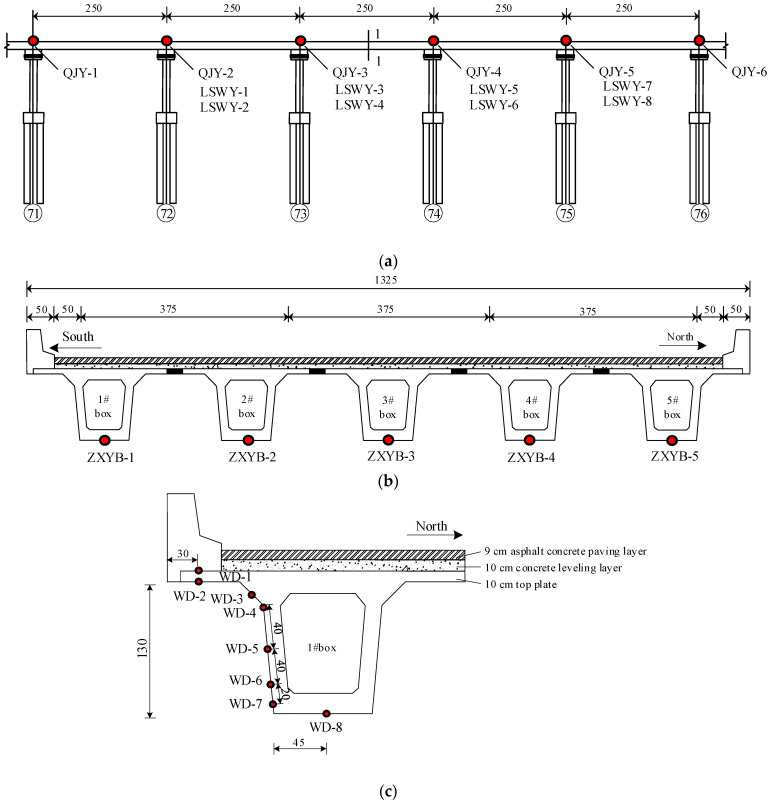
Bridge elevation and the layout of the SHM system: (**a**) bridge elevation and the layout of the inclinometers and pull-rope displacement meters (unit: dm); (**b**) the layout of the strain gauges (unit: cm); and (**c**) the layout of the temperature sensors (unit: cm).

In order to grasp the operation state of the bridge structure in real-time, five strain gauges (ZXYB-1–ZXYB-5) were arranged on the 1–1 cross-section; six inclinometers (QJY-1–QJY-6) were arranged on the 71–76# piers, and the deflection of the bridge was calculated by the rational function; eight pull-rope displacement meters (LSWY-1–LSWY-8) were arranged on the 72–74# piers to measure the pier–girder relative displacement; eight temperature sensors (WD-1–WD-8) were arranged on the 1# box of the 1–1 cross-section for real-time monitoring of the changing pattern of the temperature, and the sampling frequency of the temperature sensors was 1/600 Hz. The elevation of the bridge and the SHM system are shown in Figure 3.

### 3.2. Analysis of Monitoring Data

#### 3.2.1. Analysis of Temperature Monitoring Data

**Figure 4:**
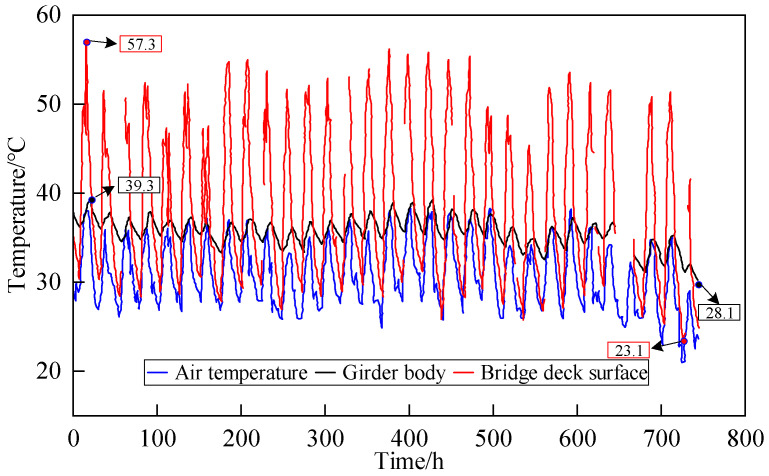
Comparison between measured points of temperature and air temperature (August 2019).

In order to analyze the temperature gradient of the cross-section, based on the location of the measurement points, the temperature measured at WD-1 was selected as the representative value for the bridge deck surface; based on the pattern of the temperature gradient [34]; the temperature measured at WD-5 was selected as the representative value for the girder body. As shown in Figure 4, it was found that the temperature monitoring results of different measurement points of the same cross-section are consistent with the trend of air temperature. The temperature of the bridge deck surface varied widely, and the temperature of the girder body varied relatively gently. Due to the different positions exposed to the environment, the extreme values of different positions are inconsistent; in August, the maximum temperature of the girder body was 39.3 °C, with a minimum of 28.1 °C; while the temperature of the bridge deck surface reached a maximum of 57.3 °C with a minimum of 23.1 °C, indicating that there was a significant vertical temperature gradient on the small box girder bridge.

#### 3.2.2. Noise Reduction for Monitoring Data

**Figure 5:**
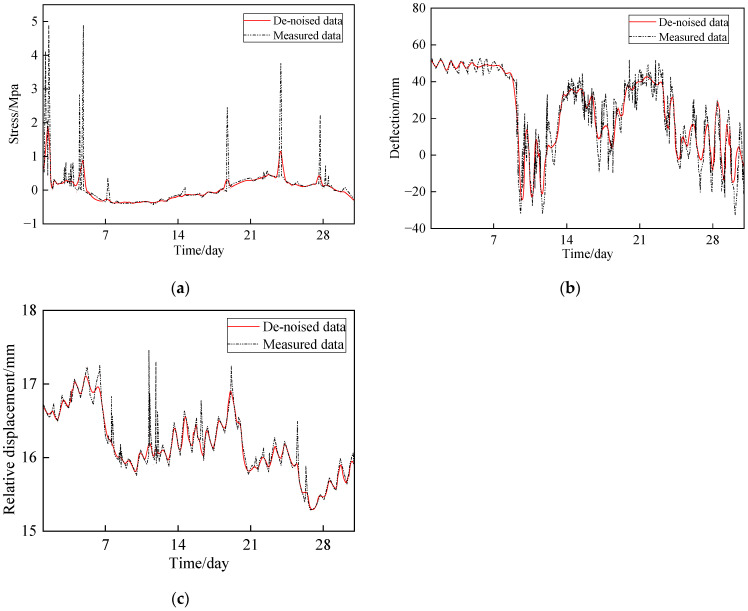
Comparison between the measured data and de-noised data (August 2019): (**a**) stress measured by ZXYB-1; (**b**) deflection measured by QJY-1; and (**c**) pier–girder relative displacement measured by LSWY-1.

In the process of bridge safety monitoring, a large amount of real-time monitoring data is an important basis for evaluating bridge safety state. To obtain accurate data, the noise interference in the original data must be removed. In this section, the Daubechies wavelet base was selected to implement the noise reduction in MATLAB. In August 2019, the stress, deformation, and pier-girder relative displacement were measured, and the measured data were subjected to noise reduction. Due to the limited space, a part of the noise reduction results for measured data is shown in Figure 5. It can be seen that the de-noised data are more stable, which is more conducive to subsequent safety evaluation analysis.

## 4. Threshold Selection

### 4.1. Temperature Gradient Threshold

In this section, the temperature field and temperature gradient of the small box girder bridge are studied based on the SHM data to provide temperature thresholds for the subsequent safety evaluation.

#### 4.1.1. The Spatio-Temporal FEM of the Temperature Field

**Figure 6:**
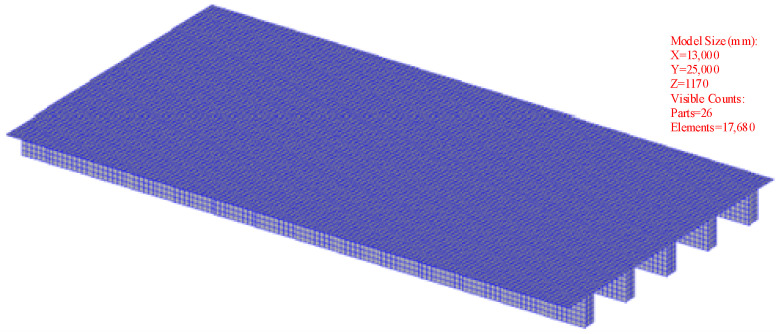
Schematic diagram of the FEM.

The FEM of the small box girder was divided into 17,680 elements. The 1st–2nd floors of the top plate are made of 4.5 cm-thick asphalt concrete, and the 3rd–6th floors are made of 5 cm-thick high-strength concrete. The 1st–6th floors of the web plate are made of 5 cm-thick high-strength concrete, the 1st–5th floors of the bottom plate are made of 3.2 cm-thick high-strength concrete, and the 1st–6th floors of the sealing cover plate are made of 1.5 cm-thick high-strength concrete. The FEM of the small box girder is shown in Figure 6.

The material thermophysical parameters and location parameters of the small box girder bridge are listed in Table 1 and Table 2, respectively. The meteorological information at the bridge site was fed into the FEM to achieve the long-term thermodynamic analysis.

**Figure 7:**
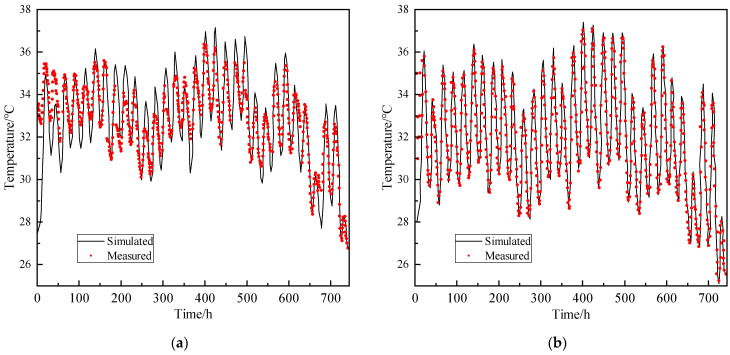
Temperature comparison between the simulated and measured value (August 2019): (**a**) top plate; (**b**) bottom plate.

The meteorological files and location information as well as the thermophysical parameters of the bridge were imported into TAITHERM. The real-time changing boundary conditions was automatically established, and then the time-range analysis of the temperature field was revealed. The SHM data of the top plate (WD-1), and the bottom plate (WD-8) in August 2019 were selected for comparison with the simulation results. It can be seen from Figure 7 that the measured temperature of the top, and bottom plates are close to the simulated values. Therefore, the established spatio-temporal FEM model can be used to study the temperature field of the small box girder bridge.

#### 4.1.2. Threshold of Temperature Gradient

Since the maximum value of the temperature gradient of the bridge cross-section usually occurs in the summer [13], the highest daily temperature of 39.70 °C in Wuhan City for the past 17 years was selected as the input for calculating the temperature gradient threshold, and the corresponding meteorological parameters were obtained from the Wuhan Municipal Bureau of Statistics. The Gumbel first extreme value distribution was used to fit the maximum temperature. According to the extreme value distribution function, a sample mean value of 38.11 °C is obtained, and the sample variance is 0.996. Considering the design reference period as 100 years, the maximum temperature is 42.13 °C [35]. The related meteorological parameters were regarded as the extreme weather conditions to form the weather documents, and then the documents were imported to the temperature-filed spatio-temporal FEM of the small box girder bridge. The calculated maximum temperature gradients of the top plate and bottom plate are 14.24 °C and 2.07 °C, respectively. The fitting equation for the most unfavorable temperature gradient can be expressed as follows:

(7) $$\left\{\begin{array}{ll} T_{y}=14.24e^{-10.12y},R^{2}=0.92 & 0\leq y\leq H-0.2\\ T_{y}=11.23y-14.84 & H-0.2\leq y\leq H \end{array}\right.$$

where *y* is the distance from the surface of the top plate; $T_{y}$ is the temperature difference at *y*; and *H* is the height of the girder. 

The specification [34] specifies the temperature gradient of the concrete box girder using a double dash form. Since the specification only provides the temperature gradient at thicknesses of 50 and 100 mm, and the asphalt concrete paving layer of the bridge in this article had a thickness of 90 mm, the temperatures at the folds were calculated using linear interpolation to be 15.2 °C and 5.74 °C, respectively.

**Figure 8:**
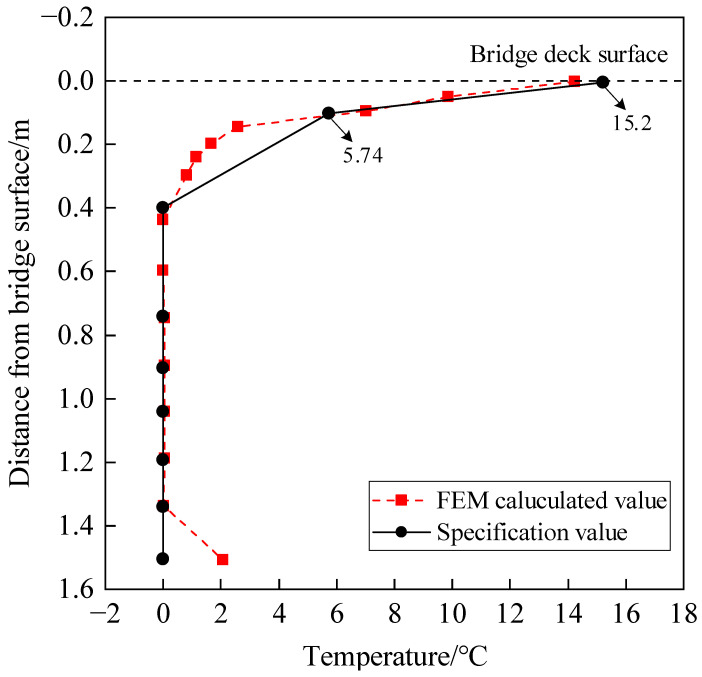
Comparison between calculated and specification temperature gradients.

**Figure 9:**
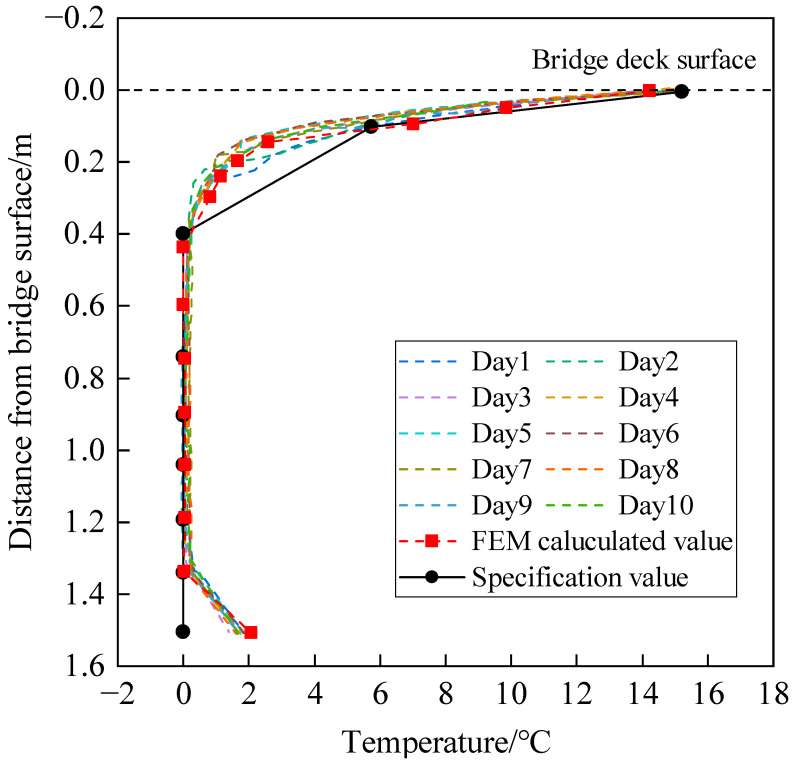
Comparison between the measured, calculated, and specification temperature gradients.

A comparison between the calculated most unfavorable temperature gradient and specification is shown in Figure 8. It can be seen that the temperature gradient of the top plate is within specification, indicating that the top plate is in a safe state. However, there is a significant difference at the bottom plate. In order to verify the applicability of the calculated most unfavorable temperature gradient, the representative most unfavorable temperature gradient of ten days from August to November 2019 measured by the SHM system is shown in Figure 9 for comparison.

It can be seen from Figure 9 that the measured most unfavorable temperature gradient agrees well with the calculated result of the FEM. Therefore, the calculated most unfavorable temperature gradient can be used as a threshold for bridge safety evaluation.

### 4.2. Thresholds of Other Indexes

In addition to the temperature index, the stress, deflection, and pier–girder relative displacement were also selected for bridge safety evaluation. The thresholds of these indexes were determined according to relative industry specifications and measured data collected by the SHM system from August to November 2019. 

#### 4.2.1. Industry Specification Threshold 

Based on the industry specifications, the thresholds of the indexes were initially determined, and the process of calculating the thresholds of these three indexes and the results are listed in Table 3.

#### 4.2.2. Historical Statistic Threshold

The historical statistical thresholds were determined by data with a 95% guarantee rate measured by the SHM system. Combining the industry specification thresholds and the historical statistical thresholds, the combined thresholds were determined. The combined safety thresholds for the indexes are listed in Table 4.

## 5. Evaluation Result of the FAHP

### 5.1. The FAHP Model

**Figure 10:**
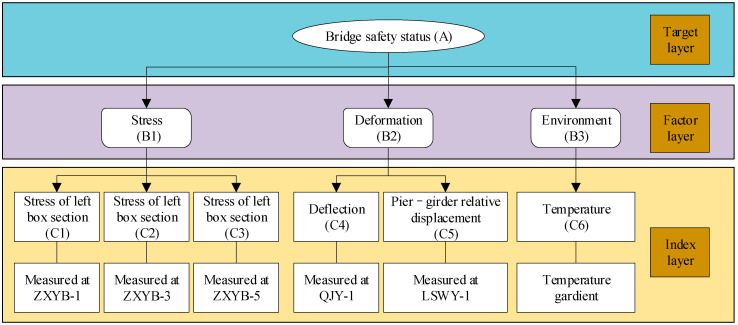
The structure of the FAHP model.

According to the relationship between bridge safety and influencing factors, the FAHP model of the small box girder bridge was divided into three levels. The top level is the target level, which indicates the working condition of the bridge. The middle layer is the factor layer, which was designed to reflect the working conditions of the top layer, and it can be divided into stress, deformation, and environment. The bottom layer is the index layer, and it is divided into stress, deflection, pier–girder relative displacement, and temperature gradient. The structure of the FAHP model is shown in Figure 10.

Without loss of generality, the stress indicators (C1–C3) corresponded to the stress monitoring results of the left girder section (ZXYB-1), the mid girder section (ZXYB-3) and the right girder section (ZXYB-5), respectively. The deformation indicators (C4–C5) corresponded to the deflection monitoring result (QJY-1) and the pier–girder relative displacement (LSWY-1), respectively.

### 5.2. Constructing the Judgment Matrix

In this article, the 9-scale method was used to obtain the judgment matrix of the small box girder bridge. By calculating the weight and the consistency coefficient, the consistency of each index was judged. 

Based on the scoring results of the expert in the bridge field, the judgment matrices **P**_AB_ between the target and factors; **P**_B1_ between the factor B1 and indexes C1–C3; and **P**_B2_ between the factor B2 and indexes C4–C5 are shown as follows:

(8) $$P_{\mathrm{AB}}=\left[\begin{array}{ccc} 0.5 & 0.6 & 0.8\\ 0.4 & 0.5 & 0.7\\ 0.2 & 0.3 & 0.5 \end{array}\right]$$

(9) $$P_{B1}=\left[\begin{array}{ccc} 0.5 & 0.4 & 0.6\\ 0.6 & 0.5 & 0.6\\ 0.4 & 0.4 & 0.5 \end{array}\right]$$

(10) $$P_{B2}=\left[\begin{array}{cc} 0.5 & 0.6\\ 0.4 & 0.5 \end{array}\right]$$

The corresponding weight vectors calculated according to Equation (2) are shown as follows:

(11) $$W_{\mathrm{AB}}=\left[0.4,0.35,0.25\right]$$

(12) $$W_{B1}=\left[0.33,0.37,0.3\right]$$

(13) $$W_{B2}=\left[0.55,0.45\right]$$

The corresponding fuzzy complementary identity matrices calculated according to Equation (3) are shown as follows:

(14) $$W_{\mathrm{AB}}^{*}=\left[\begin{array}{ccc} 0.5 & 0.53 & 0.62\\ 0.47 & 0.5 & 0.58\\ 0.38 & 0.42 & 0.5 \end{array}\right]$$

(15) $$W_{B1}^{*}=\left[\begin{array}{ccc} 0.5 & 0.48 & 0.53\\ 0.52 & 0.5 & 0.55\\ 0.47 & 0.45 & 0.5 \end{array}\right]$$

(16) $$W_{B2}^{*}=\left[\begin{array}{cc} 0.5 & 0.55\\ 0.45 & 0.5 \end{array}\right]$$

**Figure 11:**
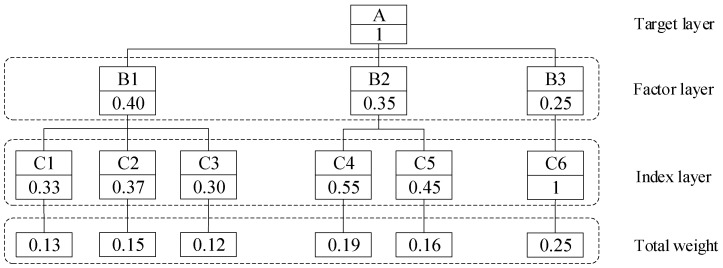
Total weight of each index.

The compatibility index of the judgment matrix with the identity matrix is 0.08 < 0.1, 0.04 < 0.1, and 0.02 < 0.1, respectively, according to Equation (5). Therefore, the weight coefficients obtained from the expert scoring are reliable and can be used for the subsequent evaluation of the bridge safety state. The total weight *W*_T_ of each index is shown in Figure 11.

### 5.3. Evaluation Matrix

To obtain the evaluation matrix of the index layer of the small box girder bridge, based on the threshold of each index, the mathematical statistics method was used to determine the specific score of each index. When the measured value exceeds the threshold interval, the measured value is taken as 1; when the measured value is within the threshold interval, take the measured value as 0; and when the measured value data are lost or changed, the measured value is −1. The parameters (C1–C6) of the index layer were counted and the negative values were not included in the total score. The score of each index is listed in Table 5.

By analyzing the score of each index, the index layer evaluation matrix of the small box girder bridge **R** can be obtained:

(17) $$R=\left[88,91,85,88,83,99\right]$$

### 5.4. Results of the FAHP

According to the index layer evaluation matrix **R** and the total weight **W**_T_ of each index in Figure 11, the fuzzy comprehensive evaluation result of the small box girder bridge can be obtained:

(18) $$Q=R\cdot W_{T}=\left[88,91,85,88,83,99\right]\cdot {\left[0.13,0.15,0.12,0.19,0.16,0.25\right]}^{T}=90.05$$

According to the specification [38], the safety state of bridges was divided into five categories. The details of each grade and corresponding interval scores are listed in Table 6.

According to the fuzzy comprehensive evaluation result, it is 90.05 ∈ [80, 95), and the bridge is judged to be a second-class bridge according to the specification. The result is consistent with the field test result, which verifies the validity and applicability of the method proposed in this article.

## 6. Conclusions

This article establishes the FEM of the temperature field and calculates the most unfavorable temperature gradient as a threshold for the safety evaluation of the bridge. Analyzing the historical statistical pattern of the SHM data of the stress, deflection, and pier–girder relative displacement, and combined with the relevant industry specifications, the threshold of each index of the small box girder bridge was determined. Scores for indexes were determined through the SHM data, and the FAHP was used to quantitatively evaluate the safety state of the bridge. The main conclusions of this article are summarized as follows:

(1) Combining ANSYS, HYPERMESH, and TAITHREM, the spatio-temporal FEM of the temperature field of the small box girder bridge is precisely established. Compared with the value given in the specification, the obtained most unfavorable temperature gradient from the FEM is closer to the actual situation, especially at the bottom plate of the small box girder bridge, and can be used as a threshold for safety evaluation.
(2) According to the historical statistical pattern of the SHM data, combined with the relative industry specifications, the thresholds of the stress, deflection, and pier–girder relative displacement are determined, respectively. The bridge safety evaluation result shows that the proposed methodology for determining the thresholds is effective.
(3) Based on the FAHP, the safety state of the bridge is quantitatively evaluated, and the weight of the indexes is determined by expert scoring. The score for each index is determined by the measured SHM data. The result of the bridge safety evaluation is consistent with the field tests, which verifies the validity and applicability of the method proposed in this article.

Although this article obtains the accurate bridge safety level, due to the limitation of the engineering background of this article, only the temperature gradient is considered as the environmental factor. When generalizing the methodology, it is suggested that bridges be equipped with a richer variety of sensors to monitor environmental factors, which in turn leads to more refined safety evaluation results.

## Figures and Tables

**Table 1:** Material thermophysical parameters.

Material	Density (kg/m^3^)	Thermal Conductivity (W/m·°C)	Heat Capacity (J/kg·K)	Reflectivity	Heat Transfer Coefficient (W/(m^2^·K))	Absorbency
Concrete	2500	1.28	970	0.85	—	0.6
asphalt pavement	2100	1.05	1168	0.93	5	0.93

**Table 2:** Location parameters of the bridge.

Longitude (°)	Latitude (°)	Time Zone (h)	Altitude (m)
−114.385	30.506	−8	44

**Table 3:** The specification thresholds of indexes.

Index	Specification	Calculation Formula	Bridge Properties	Specification Threshold
Stress	Specifications for Design of Highway Reinforced Concrete and Prestressed Concrete Bridges and Culverts (JTG 3362-2018) [36]	$\sigma_{\mathrm{tp}}\leq 0.5\cdot f_{tk}$	Made by C50 concrete	[0, 1.325] (Mpa)
Deflection	General Specifications for Design of Highway Bridges and Culverts (JTG D60-2015) [34]	$f\leq \frac{L}{600}$	Span is 25 m	[−41.000, 41.000] (mm)
Pier-girder relative displacement	Specifications for Maintenance of Highway Bridges and Culverts (JTG 5120-2021) [37]	$p_{r}\leq 5\sqrt{L\cdot 10^{-6}}$	Span is 25 m	[0, 25.000] (mm)

**Table 4:** Combined safety threshold for each index.

Index	Measuring Point	Average Value	Historical Statistic Threshold	Combined Threshold
Stress (unit: Mpa)	ZXYB-1	0.892	[−0.457, 1.327]	[−0.457, 1.327]
	ZXYB-2	0.502	[−0.916, 1.919]	[−0.916, 1.325]
	ZXYB-3	0.419	[−0.394, 1.233]	[−0.394, 1.325]
	ZXYB-4	0.488	[−0.619, 1.596]	[−0.619, 1.596]
	ZXYB-5	0.511	[−0.863, 1.885]	[−0.863, 1.885]
Deflection (unit: mm)	QJY-1	19.711	[−4.379, 43.802]	[−41.000, 43.802]
	QJY-2	37.518	[35.617, 39.418]	[−41.000, 41.000]
	QJY-3	19.723	[−2.136, 41.581]	[−41.000, 41.581]
	QJY-4	22.118	[−5.683, 49.920]	[−41.000, 49.920]
	QJY-5	31.131	[17.499, 44.764]	[−41.000, 44.764]
	QJY-6	28.463	[15.549, 41.376]	[−41.000, 41.376]
Pier-girder relative displacement (unit: mm)	LSWY-1	20.552	[15.715, 25.889]	[0, 25.889]
	LSWY-2	18.031	[12.625, 23.437]	[0, 25.000]
	LSWY-3	8.592	[−2.656, 19.840]	[−2.656, 25.000]
	LSWY-4	6.413	[−9.401, 22.226]	[−9.401, 25.000]
	LSWY-5	16.066	[7.876, 24.256]	[0, 25.000]
	LSWY-6	13.006	[−3.585, 29.596]	[−3.585, 29.596]
	LSWY-7	17.971	[9.596, 26.347]	[0, 26.347]
	LSWY-8	16.936	[8.166, 25.705]	[0, 25.705]

**Table 5:** Score of each index.

Index Layer		Count	Collection Times	Percentage	Score
Stress	C1	1513	12,847	12%	88
	C2	1152	12,847	9%	91
	C3	1866	12,847	15%	85
Deformation	C4	1921	16,101	12%	88
	C5	3670	22,191	17%	83
Environment	C6	154	14,461	1%	99

**Table 6:** Classification of bridge safety level.

Bridge Grade	Score Interval
First class	[95, 100]
Second class	[80, 95)
Third class	[60, 80)
Fourth class	[40, 60)
Fifth class	[0, 40)

## Data Availability

The data presented in this study are available upon request from the corresponding author.
